# Elevated Serum Testosterone in Young Offenders: A Case-Control Study

**DOI:** 10.7759/cureus.82370

**Published:** 2025-04-16

**Authors:** Crystal A Cadena-Molina, Carlos M Contreras-Pérez, Ana L Calderón-Garcidueñas, Ruben Ruiz-Ramos, Guadalupe Melo-Santiesteban, Noé López-Amador

**Affiliations:** 1 Forensic Medicine (National Postgraduate System of the Secretary of Science, Humanities, Technology and Innovation), University of Veracruz, Boca del Río, MEX; 2 Neuroethology, University of Veracruz, Xalapa, MEX; 3 Biomedical Research, National Autonomous University of Mexico, Mexico City, MEX; 4 Neuropathology, National Institute of Neurology and Neurosurgery, Mexico City, MEX; 5 Medicine, University of Veracruz, Veracruz, MEX; 6 Forensic Medicine, University of Veracruz, Boca del Río, MEX

**Keywords:** aggression, antisocial behavior, basal estradiol, behavioral risk factors, criminology, forensic endocrinology, impulsive behavior, juvenile offenders, progesteron, testosterone

## Abstract

Background

The role of testosterone in aggressive and antisocial behavior remains debated. While often linked to dominance and impulsivity, its causal relevance in delinquency is unclear. This study aimed to compare serum testosterone, estradiol, and progesterone levels between young male offenders and non-offending controls.

Methods

A case-control design was employed involving 28 incarcerated males (18-24 years) and 21 age-matched university students. Blood samples were collected under fasting conditions in the early morning. Free testosterone, estradiol, and progesterone were quantified via enzyme-linked immunosorbent assays (ELISA), while alanine aminotransferase (ALT) and aspartate aminotransferase (AST) levels were analyzed to assess hepatic integrity. Statistical comparisons were made using Student’s t-tests, with p < 0.05 considered significant.

Results

Plasma testosterone levels were significantly higher in offenders (14.2 ng/mL) than in controls (11.7 ng/mL; t = -2.015, p = 0.050). No group differences were observed in estradiol (t = 0.066, p = 0.947) or progesterone levels (t = 1.677, p = 0.100). ALT levels were unexpectedly elevated in the control group (59.19 IU/L vs. 39.64 IU/L; t = 2.741, p = 0.009), though AST levels were comparable (t = 0.406, p = 0.686). All hormone levels remained within clinically normal ranges.

Conclusions

Testosterone levels were elevated but clinically normal in young offenders, consistent with previous literature suggesting a potential biological contribution to aggression-related behaviors. However, hormonal profiles alone are insufficient to explain delinquency. Environmental influences, developmental history, and neural mechanisms regulating impulse control likely interact with endocrine factors. The findings support integrative models of antisocial behavior and suggest future research should combine neurobiological and psychosocial assessments. The observed ALT elevation in controls underscores the importance of strict screening in control group selection.

## Introduction

Antisocial behavior arises from a combination of sociobiological factors. Exogenous influences - such as family dynamics, deviant peer associations, socioeconomic disadvantage, poor diet, and environmental exposures - can increase the likelihood of aggression, particularly in individuals with heightened emotional reactivity [1].

Endogenous factors, particularly those related to emotional reactivity, are equally critical. Understanding these biological components may help identify biomarkers useful for diagnosis and intervention. Catecholamines, such as dopamine, norepinephrine, and serotonin, play central roles in emotional regulation and cognitive processing, and disruptions in their metabolism have been linked to aggressive and antisocial behaviors [2].

Certain psychogenetic factors also modulate aggression. For instance, Brunner syndrome-characterized by impaired impulse control and borderline intellectual disability-results from mutations in the monoamine oxidase A (MAO-A) gene. This enzyme regulates the breakdown of neurotransmitters like dopamine, norepinephrine, serotonin, and histamine. Low-expression MAO-A variants, influenced by variable number tandem repeats (VNTRs) and point mutations, may lower thresholds for emotional reactivity and impulsivity, predisposing individuals to antisocial behavior [3]. Additionally, dietary intake of biogenic and polyamines may interact with these genetic pathways, potentially altering amine levels and behavioral responses [4].

Another relevant gene is catechol-o-methyl-transferase (COMT), which encodes an enzyme responsible for degrading catecholamines in the prefrontal cortex. The Val158Met polymorphism significantly reduces enzymatic activity, resulting in increased dopamine availability. This polymorphism has been associated with heightened neuroticism, emotional dysregulation, and cognitive inflexibility [5]. These alterations may interact with androgenic signaling pathways, particularly in subcortical regions such as the hypothalamus, which integrates endocrine and behavioral responses. It has been hypothesized that genetically driven inefficiencies in catecholamine breakdown could modulate sensitivity to circulating androgens, amplifying behavioral outputs such as aggression or impulsivity. Studies combining COMT or MAO-A polymorphisms with testosterone levels have shown associations with antisocial personality traits, including elevated cerebrospinal fluid testosterone and reduced levels of 3-methoxy-4-hydroxyphenylglycol (MHFG), a metabolite of MAO activity [6].

The endocrine system's involvement in psychopathy and aggression has been explored across both clinical and experimental contexts. Elevated baseline testosterone has been associated with increased cortisol reactivity in individuals with psychopathic traits, suggesting a dysregulated stress-hormone axis [7]. In rodent models, exogenous testosterone administration has produced anxiolytic effects, while estrogen did not yield similar outcomes, indicating potential sex-steroid-specific neuromodulation [8]. Additionally, dopamine, a key modulator of reward and arousal, interacts extensively with the pituitary axis: it inhibits prolactin, suppresses gonadotropins and thyroid-stimulating hormone, and intermittently stimulates growth hormone release without altering adrenocorticotropic hormone (ACTH) levels [9]. Experimental work in Syrian hamsters has shown that anabolic androgenic steroids (AAS) increase dopamine production and D2 receptor density in the anterior hypothalamus, a region critical for aggression regulation. Antagonism of D2 receptors with eticlopride reduced aggressive behavior but induced motor side effects, highlighting the complexity of dopaminergic involvement in steroid-sensitive aggression pathways [10].

The present study aims to evaluate whether young male offenders exhibit distinct serum sex steroid profiles - specifically testosterone, estradiol, and progesterone - compared to non-offending peers. It also includes hepatic markers (aspartate aminotransferase (AST) and alanine aminotransferase (ALT)) to account for potential metabolic confounders. This case-control design provides a preliminary step toward identifying endocrine correlates of antisocial behavior. By contextualizing hormonal levels within a broader neurobiological and psychosocial framework, this research contributes to integrative models of aggression and delinquency and may guide future interdisciplinary approaches to prevention and rehabilitation.

## Materials and methods

Study design

This study employed an observational, case-control design aimed at identifying hormonal differences between young male offenders and age-matched controls. Given the constraints associated with working in forensic settings - including legal limitations and restricted access to records - the study was exploratory. Design and reporting adhered to the Strengthening the Reporting of Observational Studies in Epidemiology (STROBE) guidelines.

Ethical approval

Ethical approval was obtained from the Research Ethics Committee of the Institute of Forensic Medicine at the University of Veracruz (CEI2011-01) prior to data collection. The study complied with the principles of the Declaration of Helsinki and relevant national guidelines. Written informed consent was obtained from all participants. Given the vulnerable status of juvenile offenders, additional safeguards were implemented to protect confidentiality and minimize potential risks.

Settings and participants

The offender group consisted of 28 male participants aged 18-24 years, recruited from the State Special Detention Centre for Teenagers in Palma Sola, Veracruz. Due to legal restrictions, access to participants’ criminal records was not permitted, and clinical assessments were limited. Participants remained in the juvenile facility pending transfer to adult correctional institutions, having been initially admitted as minors. The control group comprised 21 age-matched male university students. Inclusion criteria for both groups included male sex, age between 18 and 24 years, and provision of written informed consent. Exclusion criteria were a known history of endocrine disorders, psychiatric diagnoses, or use of hormonal treatments.

Outcome and measurements

Fasting venous blood samples were collected in the early morning hours to control for circadian variability. Free testosterone was measured using a competitive enzyme-linked immunosorbent assay (ELISA). Estradiol and progesterone concentrations were determined via microparticle enzyme immunoassay. To evaluate liver function and exclude potential metabolic confounders, ALT and AST levels were also assessed using standard clinical chemistry methods.

Bias

To reduce selection and measurement bias, the study applied strict inclusion and exclusion criteria. Both groups were matched by age and sex to control for demographic confounders. All biochemical analyses were conducted under blinded laboratory conditions to avoid observer bias. However, limitations include the inability to access detailed criminal histories or socioeconomic data for the offender group, and the absence of standardized assessments for psychological or behavioral comorbidities. Environmental variables, such as diet, stress, and physical activity, were not fully controlled and are acknowledged as residual confounders.

Statistical analysis

Statistical analyses were performed using Minitab version 12.0 (Minitab, LLC, State College, PA). Between-group comparisons were conducted using Student’s t-test for independent samples. Statistical significance was set at p < 0.05. Data were reported as means ± standard error of the mean (SEM).

## Results

Participants, descriptive, and outcome data

The final sample included 49 participants: 28 young offenders and 21 control subjects. The mean age was 20.39 ± 0.36 years in the offender group and 20.04 ± 0.35 years in the control group, with no statistically significant difference (t = 0.661, p = 0.512). A non-significant trend toward greater height was observed in the control group (1.70 ± 0.013 m) compared to the offender group (1.64 ± 0.012 m; t = 2.995, p = 0.055).

Main results

As detailed in Table 1, AST levels showed no significant difference between the groups (t = 0.406, p = 0.686); however, ALT levels were significantly higher in the control group (59.19 IU/L) than in the offender group (39.64 IU/L) (t = 2.741, p = 0.009). No significant differences were found in estradiol (t = 0.066, p = 0.947) or progesterone levels (t = 1.677, p = 0.100).

**Table 1:** Biomarkers of liver function and serum estradiol and progesterone concentrations. Values are given in mean ± standard deviation (SD). ** p < 0.01, ns = not significant.

Biomarkers and hormones	Controls (n = 21)	Offenders (n = 28)	T-test	P-value
Aspartate aminotransferase (7 – 56 IU/L)	32.52 ± 3.031	31.14 ± 1.874	0.406	0.686 ns
Alanine aminotransferase (5 – 40 IU/L)	59.19 ± 7.019	39.64 ± 3.253	2.741	0.009 **
Estradiol (10 – 15 pg/dL)	14.50 ± 1.086	14.40 ± 0.965	0.066	0.947 ns
Progesterone (0.2 – 3.5 pg/dL)	1.41 ± 0.083	1.20 ± 0.091	1.677	0.100 ns

**Figure 1:**
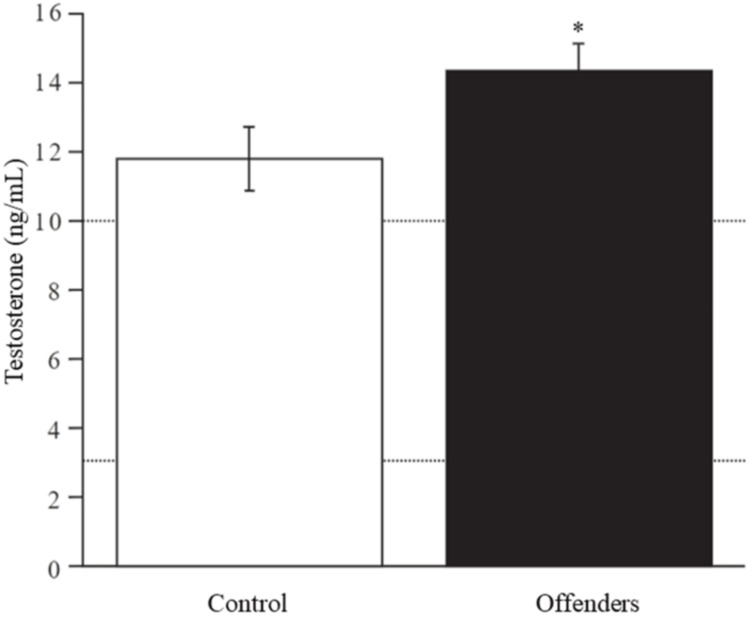
Differences in serum testosterone concentration between control and offender groups. Dotted lines represent reference values (3.5-10 ng/mL). T = -2.015, * p ≤ 0.05.

In contrast, testosterone levels were significantly elevated in the offender group (14.2 ng/mL) compared to controls (11.7 ng/mL) (t = -2.015, p = 0.050) (Figure 1).

## Discussion

Key findings

Given that steroid hormones are synthesized from cholesterol via enzymatic pathways involving hepatic metabolism, measuring transaminase activity was relevant for identifying potential confounding effects. Although ALT levels were significantly elevated in the control group, this variation is unlikely to reflect hepatic pathology due to the absence of clinical symptoms and values remaining near reference thresholds. Additionally, external variables such as physical activity, stress, and dietary differences may have influenced liver enzyme levels in the control group.

Progesterone was included in the analysis due to its documented anxiolytic and anti-aggressive properties, particularly in females [11]. It has been shown to reduce despair behaviors in female rodents [12] and modulate affective states in women, and it may reduce certain forms of aggression [13]. Although clinical studies on the role of progesterone in male aggression are limited, research in women suggests that individuals with lower irritability and aggression may have higher progesterone levels during the luteal phase [14]. In our sample, no significant differences in progesterone concentrations were found between offenders and controls, suggesting that this hormone did not meaningfully contribute to behavioral variance in either group.

Estrogens have been associated with cognition, learning, and social behavior, and also exhibit anxiolytic and antidepressant properties [15]. Additionally, they have been implicated in the modulation of certain forms of aggression [16]. In the present study, no significant differences in estrogen levels were observed between groups. This suggests that if estradiol plays a role in modulating aggression, its effects may be transient or context dependent.

Conversely, plasma testosterone levels were found to be elevated in offenders. Although levels were slightly above the clinical reference range in both groups, the difference was not indicative of underlying pathology due to the absence of clinical symptoms. Testosterone has been inversely associated with depressive symptoms [17,18] but positively correlated with aggression, violent behavior [19-21], criminal activity, and even suicide risk [22,23]. For instance, adolescents with primary gonadal disorders who received exogenous testosterone showed increases in impulsive and physical aggression [16]. Moreover, elevated testosterone has been documented among inmates convicted of violent offenses or with repeated institutional rule violations [21], prompting suggestions that testosterone may serve as a biomarker of aggression [24]. Nevertheless, elevated steroid hormone levels alone are likely necessary but not sufficient to explain aggressive conduct.

Interestingly, although testosterone is known to promote bone growth during adolescence, the control group-despite having lower testosterone levels-exhibited a non-significant trend toward greater height. This paradox may reflect the role of testosterone in accelerating epiphyseal closure via aromatization to estradiol, potentially limiting final height. Moreover, early-life variables such as nutrition, health status, and socioeconomic context - factors not measured in this study - likely contributed to these differences; other interacting neurobiological and environmental factors must be considered.

Interpretation and generalizability

The results of this study contribute to ongoing efforts to understand the biological underpinnings of antisocial behavior. The elevated testosterone levels observed in young offenders, though within the normal clinical range, support previous associations between androgens and aggression. However, hormonal profiles alone are not sufficient to explain criminal behavior. Neurobiological dysfunctions in impulse control, emotional processing, and reward systems likely interact with psychosocial stressors to shape behavioral outcomes.

While the case-control design offers valuable comparative insight, the study's generalizability is constrained by its limited sample size, specific age group, and the absence of comprehensive background data. Findings may not be extrapolated to broader populations without caution. Nonetheless, the feasibility of conducting ethically sound endocrinological research in forensic settings is demonstrated, and this work lays a foundation for future multidisciplinary investigations. These findings may inform early identification of at-risk youth and encourage integrative models for behavioral intervention that consider neuroendocrine, psychological, and social dimensions.

Aggression is a multifaceted social behavior influenced by an interplay of biological and environmental factors. Certain personality traits are closely linked to aggressive tendencies, and it is not uncommon for aggressive behavior to co-occur with substance abuse, particularly alcohol consumption [25], as well as cognitive deficits, a history of trauma, and emotional dysregulation [26]. Comparative analyses of personality profiles between individuals with a history of suicide attempts and violent offenders reveal shared characteristics, including chronic anxiety and a diminished inclination toward social interaction [27]. However, variations in specific hormone levels alone do not sufficiently account for aggressive behavior; rather, a confluence of factors, particularly prior experiences, plays a pivotal role. Furthermore, while the conversion of testosterone to estrogens has been implicated in the modulation of aggression in humans [28], testosterone has been shown to exert anxiolytic effects in female rats through mechanisms involving the γ-aminobutyric acid sub-type A (GABAA) receptor and the β-estrogen receptor, independent of its aromatization into estradiol [29]. Consequently, under normal physiological conditions, testosterone would be expected to exhibit anxiolytic properties, presenting an apparent paradox in its role in aggression regulation.

Although the precise mechanisms linking aggressive behavior to brain function remain only partially understood, evidence suggests that aggression is associated, at least in part, with reduced activity in the orbitofrontal cortex - a region critical for impulse control. Additionally, dysfunctions in neural structures responsible for emotional regulation, such as the prefrontal cortex, amygdala, and anterior cingulate cortex, have been implicated in aggressive tendencies [30]. Notably, abnormalities within the limbic, orbitofrontal, and dorsolateral prefrontal regions may play a significant role in the predisposition toward violence and the misinterpretation of emotional stimuli [25], likely interacting with a multitude of biological, environmental, and hormonal influences.

Limitations

Several limitations must be acknowledged. The sample size was relatively small, which may limit the generalizability and statistical power of the findings. Due to legal restrictions, the study lacked access to participants' full criminal records and developmental histories, which could have enriched the interpretation of hormonal profiles. Psychological assessments and measures of environmental exposures - such as early trauma, family context, and socioeconomic status - were not conducted, representing additional unmeasured confounders. Furthermore, the cross-sectional design prevents causal inference. Although fasting blood samples were collected in the morning to minimize variability, other contextual variables like diet, stress, and physical activity were not controlled. Notably, elevated ALT levels in the control group suggest the need for more rigorous health screening in non-clinical populations. Despite these constraints, the unique access to a detained youth population lends intrinsic value to the findings, offering rare biological insight into a typically underrepresented group.

## Conclusions

This study identified elevated but clinically normal testosterone levels in young male offenders compared to non-offending controls. While consistent with prior evidence linking androgens to aggression and antisocial behavior, these findings underscore that endocrine markers alone do not account for criminal conduct. A multifactorial model integrating hormonal, neurobiological, and environmental variables is necessary to understand the emergence of such behaviors. These results highlight the potential value of interdisciplinary approaches in forensic, psychological, and public health contexts and encourage further research in larger, longitudinal cohorts to refine early intervention strategies.
